# The Invertebrate Life of New Zealand: A Phylogeographic Approach

**DOI:** 10.3390/insects2030297

**Published:** 2011-07-01

**Authors:** Steven A. Trewick, Graham P. Wallis, Mary Morgan-Richards

**Affiliations:** 1 Phoenix Lab, Ecology Group, Institute of Natural Resources, Massey University, Private Bag 11-222, Palmerston North 4442, New Zealand; E-Mail: m.morgan-richards@massey.ac.nz; 2 Department of Zoology, University of Otago, P.O. Box 56, Dunedin North 9016, New Zealand; E-Mail: g.wallis@otago.ac.nz

**Keywords:** range expansion, endemicity, pliocene, pleistocene, insect, species

## Abstract

Phylogeography contributes to our knowledge of regional biotas by integrating spatial and genetic information. In New Zealand, comprising two main islands and hundreds of smaller ones, phylogeography has transformed the way we view our biology and allowed comparison with other parts of the world. Here we review studies on New Zealand terrestrial and freshwater invertebrates. We find little evidence of congruence among studies of different taxa; instead there are signatures of partitioning in many different regions and expansion in different directions. A number of studies have revealed unusually high genetic distances within putative species, and in those where other data confirm this taxonomy, the revealed phylogeographic structure contrasts with northern hemisphere continental systems. Some taxa show a signature indicative of Pliocene tectonic events encompassing land extension and mountain building, whereas others are consistent with range expansion following the last glacial maximum (LGM) of the Pleistocene. There is some indication that montane taxa are more partitioned than lowland ones, but this observation is obscured by a broad range of patterns within the sample of lowland/forest taxa. We note that several geophysical processes make similar phylogeographic predictions for the same landscape, rendering confirmation of the drivers of partitioning difficult. Future multi-gene analyses where applied to testable alternative hypotheses may help resolve further the rich evolutionary history of New Zealand's invertebrates.

## 1. Introduction

Phylogeography uses the geographic distribution of genetic variants to interpret the role of historical processes in the development of biological distributions. As originally circumscribed, phylogeography deals primarily with the structuring of populations within species [1]. This focus distinguishes it from phylogenetics and the use of species-level phylogenies to infer biogeography [2–5]. However, many of the pitfalls of biogeographic interpretation from species trees apply also to intraspecific phylogeography—there being a natural evolutionary continuum underpinning population genetics, intraspecific phylogeography, interspecific phylogeography and species phylogeny [6].

In its simplest form, two types of information can be gleaned from phylogeographic studies: first, the spatial distribution of genetic variation (e.g., whether the same DNA sequence for a gene is found in individuals from several locations) and second, the extent of differentiation between genetic variants (e.g., the proportion of nucleotide differences among DNA sequences for a given gene). Approaches founded in population genetic theory and latterly statistical phylogeography using coalescent modeling [7,8] allow description and consideration of the amount of variation present and how that variation is partitioned. Thus phylogeographic traits scale from shallow to deep in terms of divergence among sequence variants, with either intense partitioning (heterogeneous) or thorough mixing (homogenous) in terms of spatial distribution (Table 1). Many intermediary permutations are possible.

Phylogeographic analysis uses sampling in current time, but has the goal of making inferences about past populations. As with all phylogenetically-based methods, what results is a hypothesis, and the fit of the hypothesis to some other (prior) information is usually used as the basis for “testing” between alternative historic scenarios. An obvious limitation is that the method may not be able to discriminate among alternative historic processes that predict the same or similar distribution of genetic diversity. Even with the advantage of molecular clock calibration, there remains uncertainty about which extrinsic factors have influenced gene flow, because: (a) drivers may be contiguous or even coincide in time; and (b) divergence time estimates are imprecise for many reasons at the scale that is relevant in phylogeography [9–11].

### 1.1. The New Zealand Phylogeographic Context and the Development of the Fauna

**Figure 1:**
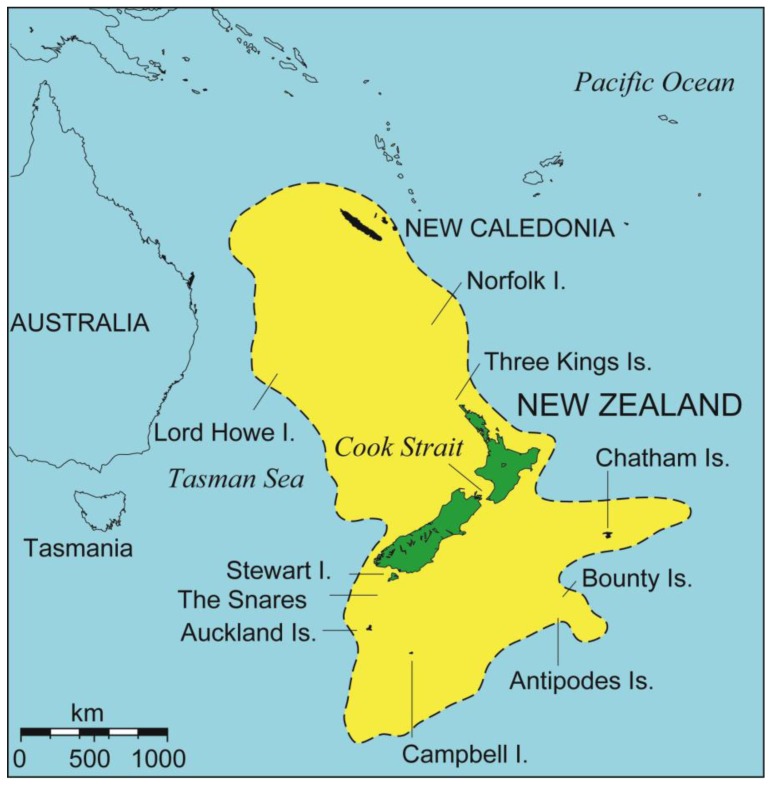
New Zealand's place in the Pacific. The approximate position of the largely submerged continental crust of Zealandia is indicated in yellow.

New Zealand is an archipelago of nearly 270,000 km^2^ situated in the south-western Pacific Ocean [12] (Figure 1). There are two main islands separated by a narrow seaway, Cook Strait, with a much greater distance of ocean to other significant land areas. Australia is a minimum of 1500 km to the west and the island of New Caledonia is 1500 km north. There are a number of small island groups within New Zealand waters including Chatham (east), Three Kings (north) and subantarctic (south).

**Figure 2:**
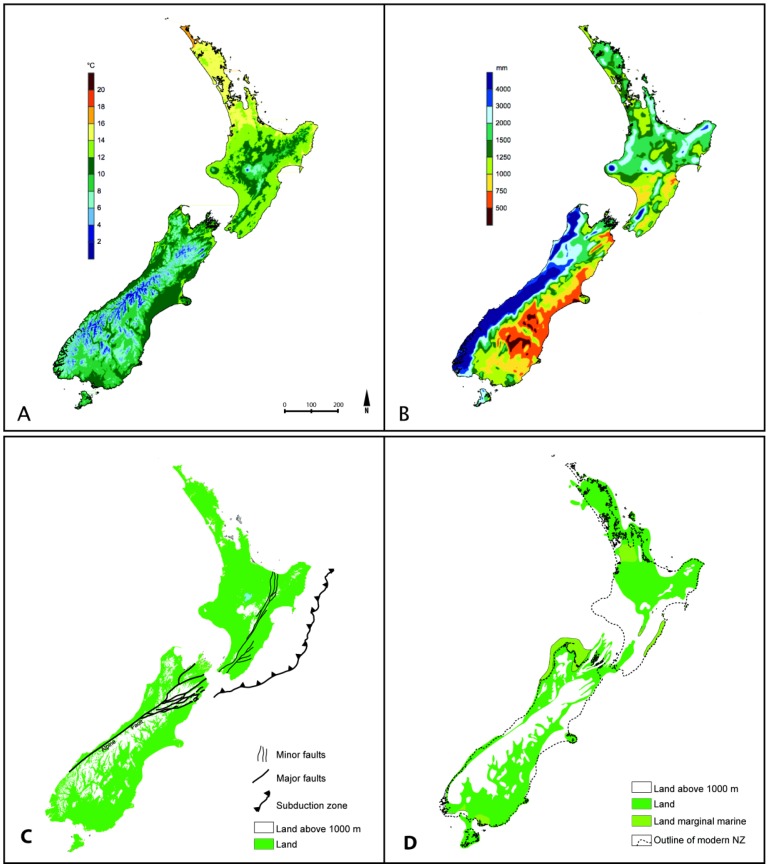

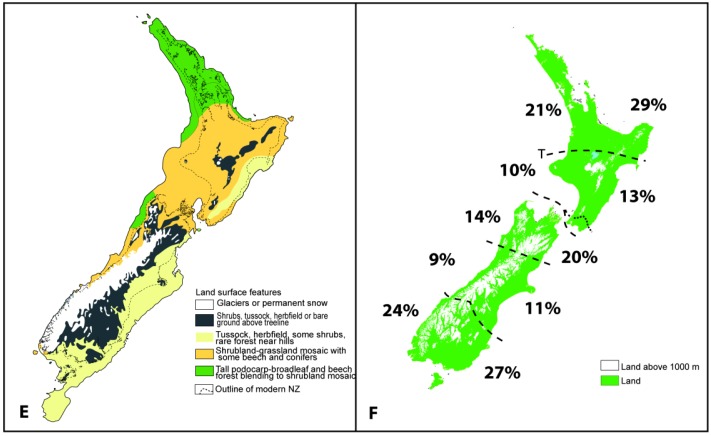
Geophysical and biogeographic features of New Zealand past and present. Environmental heterogeneity: **A**, mean annual temperature, **B**, mean annual rainfall, **C**, elevation. Temporal changes: **D**, Pliocene palaeogeography at 3 Ma, **E**, Pleistocene LGM, may yield uneven distribution of biodiversity (**F**). **F**, regional insect endemicity in a sample of 1724 species, % of species in a region that are endemic to that region (left), % of all 596 regional endemics that are endemic to a particular region (right). Thus phylogeographic (population) structure is a product of current and past environmental structure. Climate maps (**A**, **B**), courtesy of NIWA [91]. Palaeogeographic reconstructions (**D**, **E**) based on [92] and [93] respectively. Regional insect endemicity (**F**) from analysis of data in Fauna of New Zealand series volumes (2,3,12,15, 16,20–21,23,25,27,30,3–36,39–50,53,54,57–59,62,63,65) containing suitable information.

Uncertainty about the history of the New Zealand landscape (neither certainly continental nor oceanic [13]), and the presence of some peculiar elements in the fauna, have led biologists to view the New Zealand biota as extraordinary, even “the nearest approach to life on another planet” [14]. However, ecologically and morphologically distinct species are also a common feature [15] of islands much younger than the widely-assumed antiquity of New Zealand (e.g., 70 million years [16]). While plate tectonics and the breakup of Gondwana underpin the early physical development of the region, recent analyses have emphasized the onset of continental plate boundary activity at the start of the Miocene (∼25 Ma), in formation of New Zealand [17–19]. Most if not all of the pre-existing continent of Zealandia, which is an order of magnitude larger than New Zealand, is today below the sea [21,22] (Figure 1). A more youthful perspective on New Zealand geology has enabled biological thinking to move away from a focus on ancient isolation and toward evolutionary dynamism [5,22–27]. Even since Pliocene time (∼5 Ma), New Zealand has experienced extensive mountain building (Southern Alps), land extension (southern North Island), volcanism (Taupo Volcanic Zone), and climate/land changes associated with Pleistocene glaciations [20,28] (Figure 2).

As in most parts of the world, the New Zealand fauna is dominated by invertebrates and in particular insects (∼60% of 34,636 recorded New Zealand Animalia are arthropods [29]). This situation is emphasized by deficiencies in the extant native biota of several vertebrate groups (no snakes, no terrestrial mammals) [26,30,31], although among invertebrates too, a number of groups are underrepresented (e.g., within Orthoptera there are no native Gryllacrididae, and few Gryllidae and Tettigoniidae). As would be expected from its location, New Zealand fauna has its closest affinity with animals in other southern hemisphere lands. This southern distribution pattern is often referred to as “Gondwanan”, but this term can be confusing as it confounds current distribution pattern with a particular process that might have created it (namely plate tectonic breakup of Gondwana) [23]. It is increasingly recognized that the presence of “Gondwanan” lineages in New Zealand provides only limited information about the biogeographic process in development of the biota; vicariant and dispersal hypotheses often yield similar distribution patterns [23,32]. To what degree presence, absence, diversity of lineages and ecological traits are reliable indicators of the timing and extent of past occupation, arrival and extinction, is unclear and often difficult to ascertain [27]. What is more readily achieved is an understanding of the way current diversification has developed within extant lineages. In the last decade or so, a rapid expansion in the number of taxa that have been subject to some type of phylogeographic analysis has transformed our knowledge (Tables 2 and 3; see also [26]). While initial studies were largely descriptive of spatial patterns inferred from the partitioning of genetic data, they resulted in the development of hypotheses about past evolutionary processes in New Zealand invertebrate biology. They have also informed our understanding of biodiversity, systematics, taxonomy and conservation status, and enabled empirical comparison with biota in other parts of the world.

### 1.2. Predicting the Past

**Figure 3:**
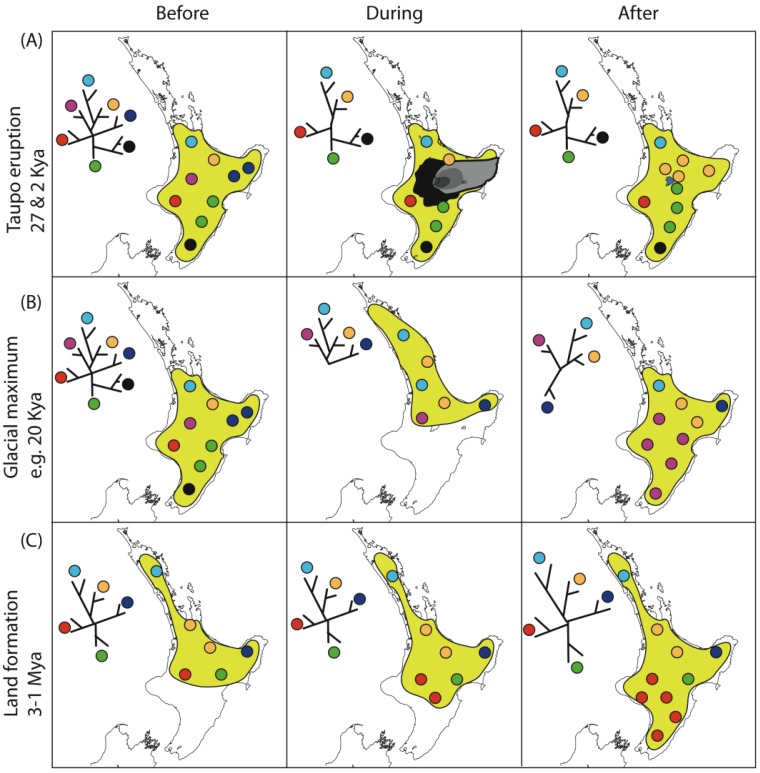
Phylogeographic outcomes of different geophysical events in North Island New Zealand may be similar. (**A**) Taupo volcanic; (**B**) LGM forest range; (**C**) land emergence since 2 million years ago. Yellow area indicates range of hypothetical taxon. (**A**) Black and grey indicate area affected by pyroclastic flow and ash deposits from Taupo eruption. Existing diversity, which may or may not be partitioned in space is extinguished close to centre and subsequently replaced by range expansion. This is expected to result in reduced diversity around the centre; (**B**) Climate cooling during glacial events resulted in retraction of forest northwards, and formation of potential refugium. Subsequent expansion of habitat is expected to result in lower diversity in south compared to north through leading-edge re-colonization; (**C**) A near identical phylogeographic pattern is expected to result from land formation which resulted in southward extension of North Island, but branch lengths may be greater than B and might be associated with taxonomic subdivision. Sequential events in the same region might yield a wide number of permutations in different taxa reflecting ecological or stochastic processes.

**Figure 4:**
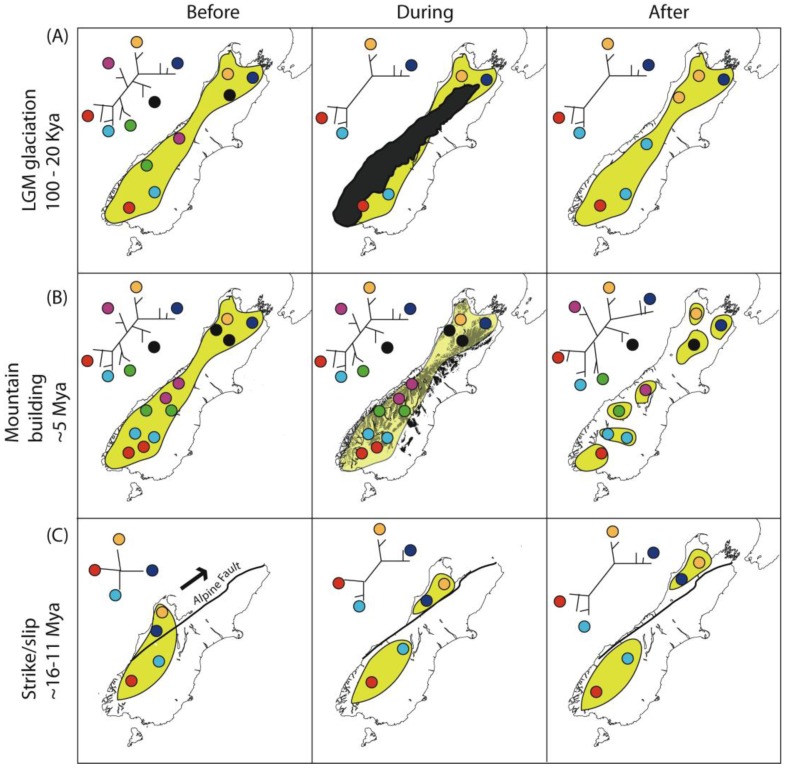
South Island (**A**) Habitat partitioning by glaciation, (**B**) Formation of alps, (**C**) Alpine fault displacement. On a long narrow island a widely distributed taxon is likely to develop a pattern of isolation by distance, even without any habitat heterogeneity. Geophysical processes may influence the gene genealogy among populations and species that evolve. Yellow area indicates range of hypothetical taxon. (**A**) Glaciation (black area) might cause extinction of some populations (and their genetic lineages), and partition residual populations in the north and south. Subsequent retraction of glaciers could allow expansion of forest taxa through leading-edge colonization; (**B**) Formation of alps (black areas) might yield habitat heterogeneity and reduce gene flow among populations leading to formation of allopatric species; (**C**) Alpine fault displacement (alpine fault line in black) might sunder adjacent populations enabling their independent evolution over time, resulting in similar phylogeographic structure resulting from lineage extinction A.

**Figure 5:**
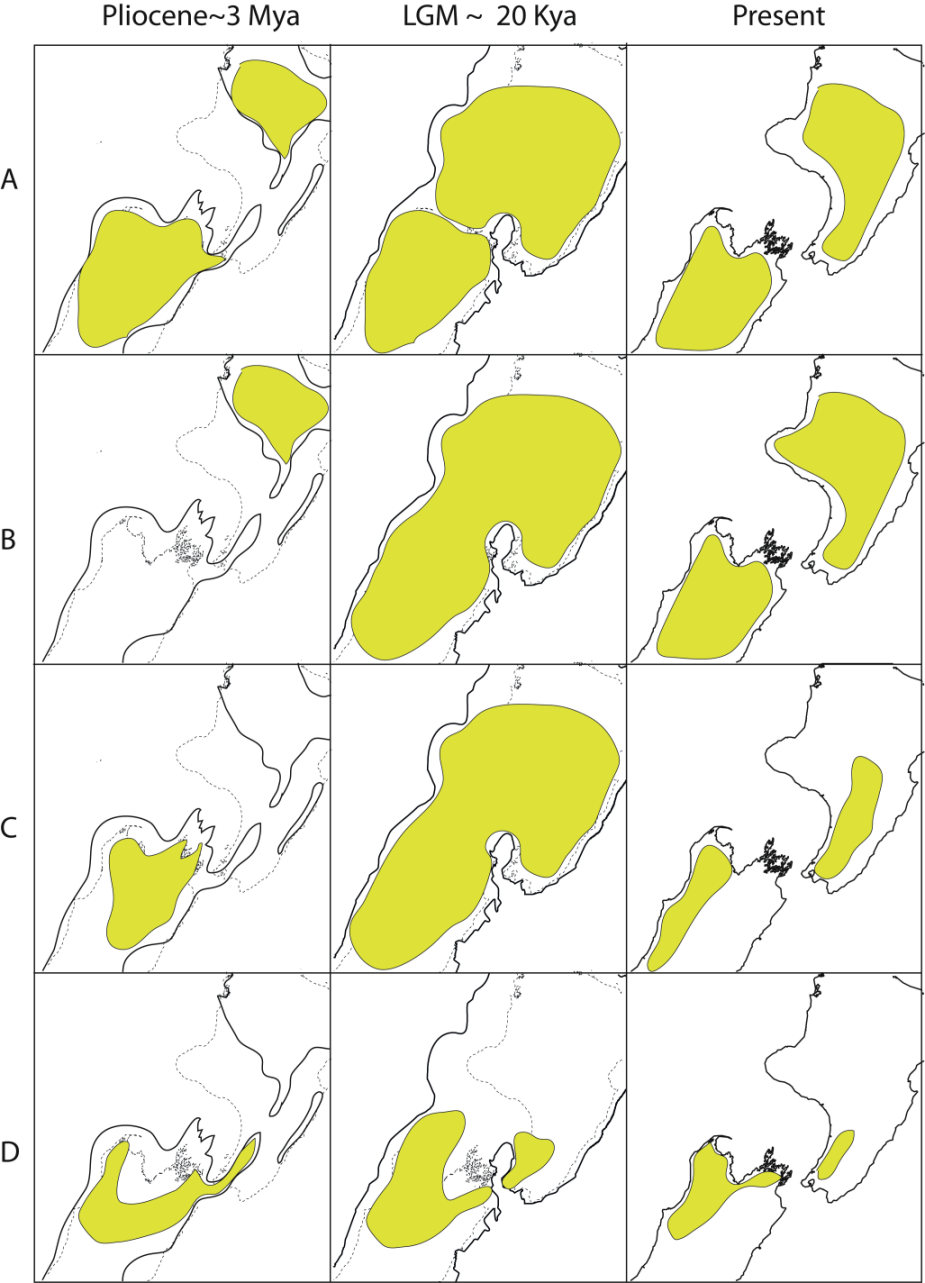
Cook Strait connections. A wide range of scenarios for South Island-North Island phylogeography are plausible. Yellow area indicates range of hypothetical taxon. (**A**) Population might be allopatric on older islands having moved between by oversea dispersal, before coming parapatric during LGM and so remaining specific to different islands; (**B**) Taxon might exist on one island only, expanding its range during LGM and then being partitioned as sea-level rises; (**C**) Taxon might initially be restricted to alpine environment on one island and colonizing new alpine environment when able during extension of lowered alpine habitat in LGM; (**D**) Taxon range in former South Island might result in occupation of southern North Island as it forms before LGM.

Spatial variation in climate, topography and vegetation generates fairly steep north-south, and in some places east-west gradients (Figure 2). Spatial variation intersects with temporal variation in these features, as changes in land area have been considerable in New Zealand during and since Pliocene time (Figure 2) [92,94]. Together, these processes have resulted in a stark pattern of regional diversity. This diversity, more usually expressed in terms of regional endemicity, was first notably documented in plants [95], but is also evident among insect groups (Figure 2E). More recent geophysical changes have probably erased large amounts of older biodiversity and population structure [92]. For example, the effect of volcanic activity in the central North Island Taupo Volcanic Zone less than 2000 years ago may have obscured population structure of forest species that had accumulated since the last glacial maximum (LGM ∼20,000 years ago), and a larger volcanic eruption in the same place 27,000 years ago may have overwritten the population structure that accumulated in North Island during the previous Pleistocene interglacials (Figure 3A). Not only are more recent geological and climatic events likely to obscure earlier patterns, but different events can sometimes leave similar spatial patterns of genetic diversity (Figures 3–5). For example, the effects of southward extension of forest habitat after the LGM (Figure 3B) could result in similar distribution of diversity as earlier southward extension of land in North Island (Figure 3C). Similarly, in South Island, low genetic diversity in a central swathe resulting from extinction of lineages yields a pattern similar to vicariance models involving glaciation or alpine fault movement (Figure 4) [49,96]. Where alternative process generate similar or identical tree hypotheses, comparison of tree topology will not distinguish them, but branch lengths can in principle exclude alternatives. Such an approach is, however, dependent on the availability of suitable molecular clock calibrations, sufficient precision for the time frame of interest, and consistency among genes and lineages. All these attributes are problematic for most phylogeographic studies, but emerging genome scale data, and coalescent model based approaches promise to enhance the sensitivity of phylogeographic testing [7,97,98].

### 1.3. Pattern and Process

Identifying the pattern of genetic structuring among populations (or other samples) is just a first step in phylogeography, as the main motivation of such work is to inform on the nature and timing of extrinsic processes that have driven population structuring. Despite a traditional focus in New Zealand biogeography on ancient processes (e.g., Gondwanan breakup, land reduction during the Oligocene), most if not all species-level diversity is much younger and coincides with the geophysically active period during the Plio-Pleistocene (Figure 2). As phylogeography deals with contemporary samples, we can be more confident about the influence of events that occurred recently than that of earlier events, because the former will tend to obscure the signal from the latter.

**Figure 6:**
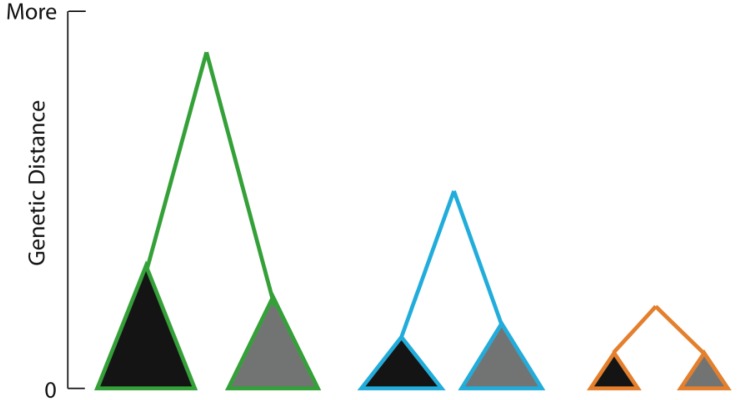
Three hypothetical taxa (green, blue, gold), each with populations in the same two areas (grey, black). Coalescent depth may differ among them, but this might be due to population size, gene/taxon specific mutation rate, lineage sorting effects or timing of historical event. Distinguishing between events requires clear statements of assumptions made in dating of nodes, to allow testing among alternative drivers of population partitioning if circularity is to be avoided [7].

A general expectation in biogeography and phylogeography is that organisms occupying the same spatial range are likely to have been subject to the same evolutionary drivers and this should be evident in their similar phylogeographic signatures [1,99–101]. It has, however, often transpired that regional phylogeographic structure is only broadly concordant and that different types of plants and animals respond in different ways [26]. One complication is that different taxon groups may sample different (spatially overlapping) episodes in history. In addition, ecological traits (e.g., fecundity, dispersal behaviour) are likely to influence population structuring. Thus, similar patterns of spatial partitioning within different species may reflect distinct temporal episodes that have operated across the same geographic space. Only comparison of genetic distance data as an estimator of time (with caution) might enable their distinction (Figure 6).

### 1.4. Sampling for Phylogeography

The scope of studies of New Zealand invertebrates that involve some spatial and genetic information is enormous, ranging from multispecies to single species, widely sampled to narrowly sampled, with shallow genetic diversity to variation at the informative limits of cytochrome oxidase I (COI) sequencing (Table 4).

More than 50 studies deal with species-level variation, usually with relatively small samples of individuals from several species (Table 2), and have been used to infer processes ranging in age from 2000 years ago until the Miocene. For almost all the patterns observed there are invertebrate examples that show contrasting patterns, revealing the complexity of New Zealand's geological history and the ecological variety sampled. We include 37 population-level phylogeographic studies of invertebrates, of which 28 studies are of insects (Table 3). These latter papers report mtDNA sequence data from individuals of (putatively) single species sampled at multiple locations. More than half these studies also include some data representing the nuclear genome including morphology, song, allozymes, cytogenetics and DNA sequences of nuclear genes. Nuclear gene sequences can provide additional gene genealogies to inform phylogeographic analysis [102], and this has contributed to some New Zealand studies [39,60,68,79,104–106]. However, nuclear markers of some form are also essential for identification of cryptic species, or conversely, confirmation that samples are from conspecifics. For example, inclusion of allozyme data provided confidence that cryptic species were sampled in the amphipod *Paracalliope fluviatilis* [107] and morphologically-conservative peripatus *Peripatoides novaezealandiae* [108]. In contrast, chromosome and allozyme analyses of the tree weta *Hemideina thoracica* confirmed that cryptic species were not present [61,109] and challenged the status of two species of *Prodontria* [65]. High mtDNA diversity within the fungus beetles *Pristoderus bakewelli* and *Epistranus lawsoni* [51], and koura (freshwater crayfish) *Paranephrops zealandicus* [47] await further analysis to determine whether reproductive isolation is likely. The mitochondrial genome is haploid and maternally inherited and therefore it cannot be used to delimit species boundaries. Without such confirmation, spurious inferences about high intraspecific mtDNA diversity or the existence of cryptic species are likely, and this undermines the power of data to test among alternative hypotheses about phylogeographic process, timing and outcome (Figures 3 and 4).

Taxon selection in phylogeographic studies tends to be biased toward widespread species, because these are perceived as having the potential to provide data about large-scale phenomena. Widespread species are more likely to be chosen because their ranges will more often encompass geophysical features of interest. These species by their very nature, however, are more likely to have high gene flow and connectedness [110], and may not be representative of a tendency in other lineages to evolve reproductive isolation mechanisms or morphologically distinct units. In contrast, lineages that have speciated extensively (e.g., stoneflies [39]; cockroaches [49,76]; cicada [60,72,73,79]; peripatus [37,49,67,108] tend not be used for the same kind of study. Interspecific studies might provide useful insights into historical processes in evolution where species are primarily allopatric or parapatric [9,49,60]. Expansion of species' ranges into sympatry will tend to obscure biogeographic patterns and conceal the historical drivers of lineage formation [108], but where interactions are limited it remains possible to estimate and test evolutionary scenarios [70] (Table 2).

## 2. Refugia and Expansion

Variation in the level of mtDNA diversity may by used to infer population expansion from an area of high diversity towards one of low diversity [111]. Half of the New Zealand intraspecific phylogeographic studies show a genetic pattern from which range expansion is inferred (18 of 37; Table 3). Source areas of range expansion (putative refugia), usually related to post-glacial climate change, that have been inferred from phylogeography include northern North Island, southern South Island and northern South Island (references in Table 3).

We note from an inventory of regional New Zealand insect endemicity (Figure 2F) that diversity is not homogenous or even graded across latitude. Instead, areas of relatively high endemicity exist in the northern North Island, northern South Island and southern South Island, similar to that identified in plants [95]. Although there is some correspondence between areas of high endemicity and location of phylogeographic refugia, the relationship between these different data has yet to be explained. Probably they relate to instances of speciation, range expansion and extinction in quite different time frames.

With only 37 independent intraspecific phylogeographic studies of invertebrates in New Zealand we find few statistically supported patterns although trends are revealing. Forest (n = 17) and aquatic (n = 11) species show all manner of phylogeographic patterns (Table 3). In contrast, species sampled from subalpine, montane and open habitats (n = 9) show a high proportion with intense regional differentiation; only two such species showing genetic evidence of “expansion”. This is consistent with the idea that topographical heterogeneity facilitates partitioning. Winged species (n = 12) are not more prevalent among the examples of range expansion as might be expected of animals capable of flight, though they do contribute relatively few studies (17%) with maximum intraspecific pairwise distances above 0.10. Species with high intraspecific pairwise distances come from all environments, but are slightly more common in forest species (41%). In six of the seven forest species where high intraspecific pairwise distances were encountered this may be partially explained by a high likelihood of cryptic species being sampled in contiguous/homogenous habitat. Conversely, a relative paucity of high intraspecific genetic distances among taxa in heterogenous environments (e.g., alpine) may be because allopatric population partitioning corresponds more frequently with species taxonomy. Fewer than half the studies tested for isolation-by-distance (IBD), but of those that did, 11 showed a positive relationship between genetic and geographic distance, and five did not. Interestingly, lack of IBD occurs in species with both small COI divergences (e.g., *Acanthoxyla*; 0.022 uncorrected) and large (e.g., *P. bakewell*; 0.149 uncorrected).

**Figure 7:**
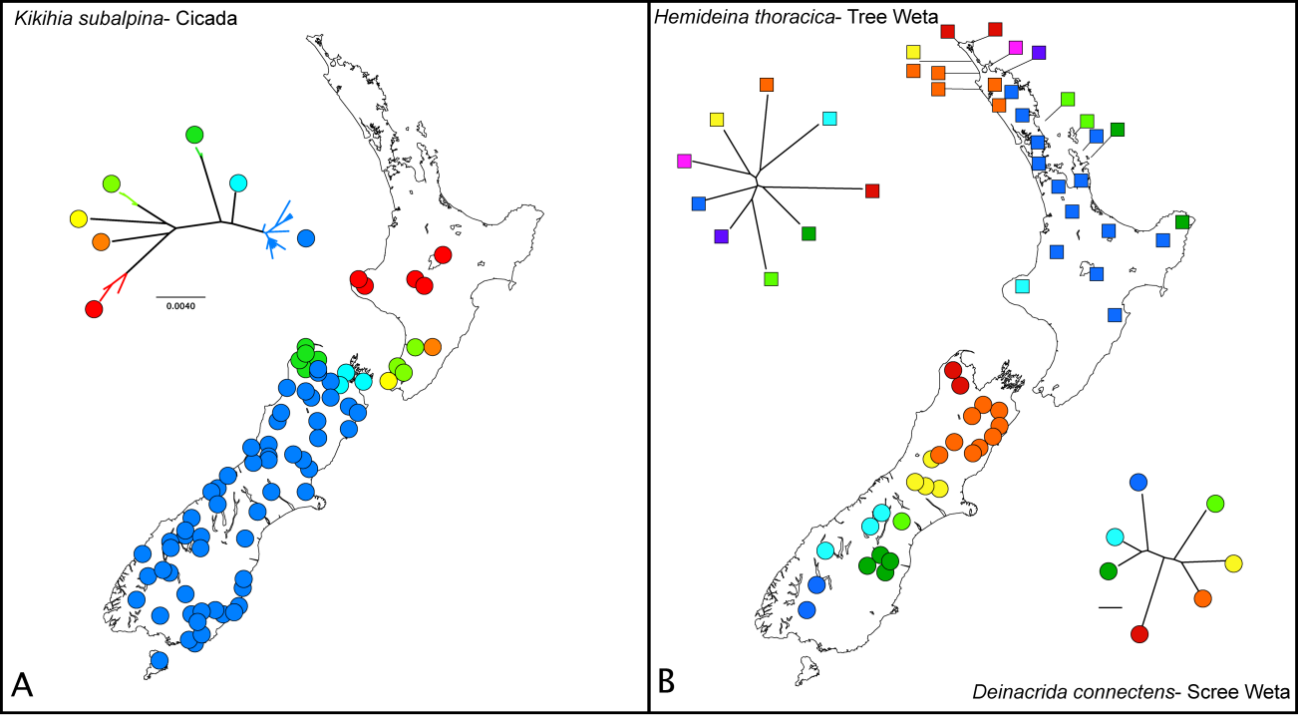

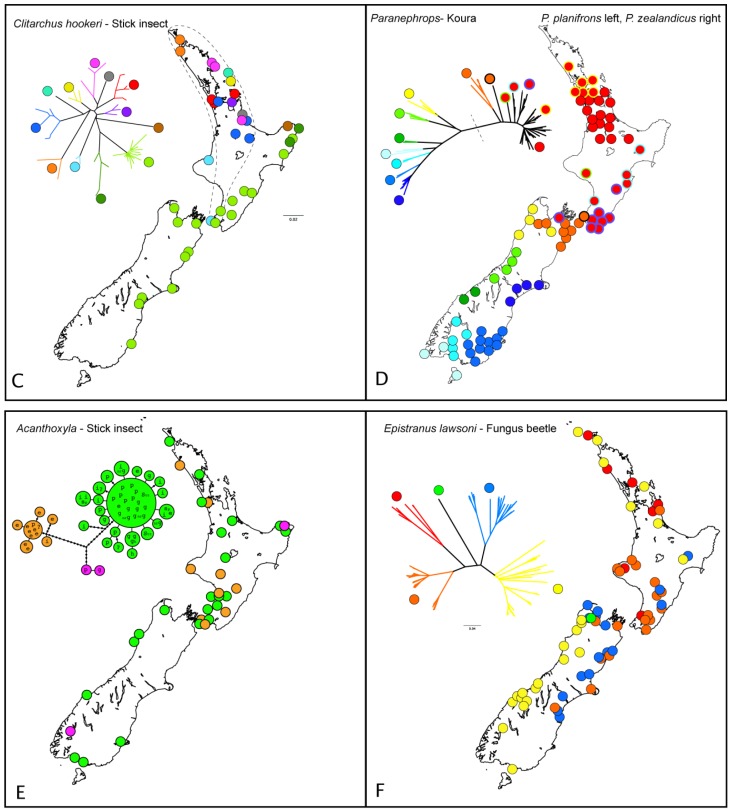
Exemplars of phylogeographic patterns revealed in species of New Zealand invertebrates. In each case mtDNA lineages are color coded and mapped, with cooler colors (blue, green) to south and warmer ones (reds) to north. Trees for A, C and F inferred using Neighbor-Joining of HKY distances in Geneious Pro v5.3.4 with mtDNA COI sequences download from GenBank; tree topology did not differ significantly from that originally reported. (**A**) Cicada *Kikihia subalpina* [59]; (**B**) Tree weta *Hemideina thoracica* only major mtDNA lineages for each species are indicated here [61], scree weta *Deinacrida connectens* [37]; (**C**) Within the stick insect *Clitarchus hookeri* [62], lineages associated with sexual populations are multicolored within dashed line whereas green populations are parthenogenetic; (**D**) The two New Zealand koura or freshwater crayfish *Paranephrops* are partitioned north and south, tree redrawn as above [47]; (**E**) Maximum parsimony network of the stick insect genus *Acanthoxyla* comprises numerous parthenogenetic morphospecies: e *A. inermis*, P *A. prasina*, i *A. intermedia*, g *A. geisovii*, Sp *A. speciosa*, Stt *A. suteri*, nrg *A.* nr *geisovii* (Myers *et al.* subm). Refer to Table 2 for data details; (**F**) The fungus beetle *Epistranus lawsoni* is likely to include cryptic species, tree redrawn as above [51]

Patterns such as “out-of-north” almost certainly result from more than one process. For example, cicada, snail, caddisfly, mayfly and stick insects (*Kikihia subalpina*, *Potamopyrgus antipodarum*, *Orthopsyche fimbriata*, *Acanthophlebia cruentata*, *Clitarchus hookeri*, *Argosarchus horridus*) might have expanded their ranges southward in a similar and relatively recent time-frame as maximum mtDNA distances within each are similar and relatively small (3–4.7%; Table 3, Figure 7). A common inference in such cases is that these taxa were influenced by Pleistocene climatic cycling (see Figure 2). However, it is unlikely (assuming population size and rate of molecular evolution are similar- Figure 6) that mite-harvestman (*Aoraki denticulata*) and tree weta (*Hemideina crassidens* and *H. thoracica*) were expanding southwards at the same time, as they show quite different intraspecific pairwise mtDNA distances (19.2, 12.7 and 9.5% respectively). A refugium (at some undefined time in the past) in the Nelson region (northern South Island) is possible for centipedes (mtDNA genetic distances up to 32%), koura (13.5%; Figure 7), mite-harvestman (19.2%), tree weta (12.7%), and fungus beetles (24.6%). As the processes involved are likely to be different (based on the range of genetic and taxonomic/ecological diversity sampled) it can be misleading to identify similar patterns. Furthermore, most New Zealand phylogeographic studies find a number of patterns and inferred processes [70] within a single taxon (Table 3).

## 3. Gaps and Regional

From the 37 putative population-level studies we can conclude that many invertebrates were apparently unaffected by the Pleistocene LGM, as they have widespread, high genetic diversity (e.g., fungus beetles *Epistranus lawsoni* and *Pristoderus bakewelli*, ground weta *Hemiandrus maculifrons* and *H. pallitarsis*, scree weta *Deinacrida connectens*, mite harvestman *Aoraki denticulata*, stick insect *Niveaphasma annulata*, stonefly *Zelandoperla fenestrata*, waterboatman *Sigara potamius*) (Table 3). For example, we see regional diversity and widespread sympatry of divergent haplotype lineages in the forest (fungus beetle Figure 7F). There is clearly differentiated regional diversity in alpine (weta Figure 7B), aquatic (stonefly) and open grass/scrub taxa (cicada Figure 7A). Species that are likely to have extended their ranges during cold glacial cycles, such as alpine, sub-alpine and open- habitat species are well sampled in the New Zealand phylogeographic literature (Table 2). These taxa show regional variation, distinguishing populations that, although currently isolated, could have been connected at lower altitudes during colder times. Alpine environments are thought to have first appeared about five million years ago when fault movement started the formation of the Southern Alps. Evidence of multiple origins of alpine adaptation comes from studies of weta [80] and flightless scarabaeid beetles [65]. The formation of the alpine zone resulted in species radiations (e.g., spiders, moths, cicada, cockroaches, grasshoppers) and the origin of intraspecific diversity (e.g., scree weta, cicada). In contrast, a few insects with low diversity and little geographic structure might be the product of recent population expansion post LGM, such as the lineage of parthenogenetic stick insects *Acanthoxyla* that have arisen via hybridisation (Figure 7E), and the damselfly *Xanthocnemis zealandica*.

The most obvious gap in terrestrial habitat in mainland New Zealand is Cook Strait, a narrow seaway (minimum 25 km) between North Island and South Island [92]. This feature corresponds approximately with one margin of an area of relatively low endemicity in southern North Island among plants and insects (Figure 2F) [95,119]. This current division of North and South Islands is no older than 500 ky [120], and was almost certainly bridged during the LGM when sea level dropped (Figure 2E) [121]. Although two main islands have probably existed at least since the late Miocene, the position and extent of seaways between them has changed over time with a general southward migration of a wave of uplift and subsidence [92]. Eight phylogeographic studies of invertebrate species reveal individuals with similar or identical haplotypes either side of Cook Strait, while six show evidence of a phylogeographic break there (Table 2). While sharing of similar haplotypes across the Strait must indicate recent gene flow (either by over-sea dispersal or LGM landbridge), partitioning could be the result of anything from recent lineage sorting via small populations (a lower level of gene flow) to clade formation on islands older than Cook Strait with their current close proximity resulting from land emergence in southern North Island (see Figures 3 and 5). Numerous genealogical histories are consistent with the formation of Cook Strait, but few if any may have originated as a result of it. Range expansion during LGM, or species ranges that were spanning old North Island and South Island, or species ranges with a connection to southern tip of North Island during Pliocene, all make subtly different genealogical predictions, the relative probability of which might be estimated in future using multigene phylogeography (Figure 5).

## 4. Dispersal Ability

With their flighted and flightless forms, insects display a wide range of dispersal capability and behaviour. Flightlessness evolves quickly and repeatedly, even showing polymorphism within species [122], presenting opportunities to examine its effect on phylogeographic structure on an otherwise closely related genetic background. It is often assumed that capacity for dispersal is readily inferred from presence of wings, but many factors might influence dispersal ability [56,77]. A comparison of phylogeographic structure among macropterous, micropterous and apterous forms of the stonefly *Zelandoperla fenestrata*, found all three to be quite highly structured, consistent with the field observation that the winged form is rarely, if ever, seen in flight [105]. In contrast, the stronger flying *Z. decorata* showed much less structure. In a cicada, lineages are finely subdivided, despite the cicada being widespread and apparently a good flier [89]. Studies of insect phylogeography encompassing New Zealand and its offshore islands are revealing in this respect. In particular, data for Chatham Islands fauna provide compelling evidence that dispersal can take many forms and is not necessarily linked to presence of wings [23]. Successful migrants to the Chatham Islands [56] include damsel flies [88], isopod [57], cicada [89], wingless beetles and crickets [77], beetles [51] and worms [54].

## 5. Partitioning and Pruning

A hypothesized effect of glaciation in South Island is vicariance (division) of widespread species into isolated populations [39]. Glaciation tends to sunder populations allowing their independent evolution (vicariance), but may also prune out intervening diversity non-randomly. The net result is phylogenetic trees with long internal branches that correspond to widely spaced and often disjunct distributions in the modern fauna (Figure 4), and such patterns are commonly reported in intra and interspecific phylogeographic studies of taxa with north-south disjunct distributions in South Island [39,49] (Table 2). Vicariance may have little to do with lineage formation in such cases, and pruning will tend to push back the inferred age of origin of surviving lineages if this is not taken into account [5]. Pleistocene glaciation consisted of a series of some 20 potential isolation/pruning episodes, and it is possible that deeper splits in phylogenetic trees were related to Pliocene diversification during mountain building [69].

## 6. Conclusions

The current data indicate that all manner of phylogeographic patterns are identifiable among New Zealand invertebrate taxa (Table 4), sustaining inferences of diverse geophysical events in their evolution. Although it is well established that phylogenies are gene trees, the phylogeographic and systematics literature shows continual lapses into treating them as taxon trees. Similarly, the associated inference of particular historic processes in genetic partitioning that are in fact only weakly consistent with the data are common-place. The emergence and availability of next generation sequencing will be a boon to phylogeography: it will become commonplace to use multiple nuclear markers and differences in these gene histories will continually remind us of their fickle reflection of the taxon trees, of which they are but a fragment. Equally, there are challenges, not least in how we use these clouds of gene trees to estimate phylogeographic history [123–125].

Even with the best multigene data to infer phylogeographic patterns, it will remain difficult to distinguish among some events that might be drivers of population partitioning and speciation in New Zealand. The geophysical events that have shaped New Zealand often overlap in their temporal influence, so future phylogeographic studies should concentrate on alternative hypotheses that can be distinguished, such as Pliocene *versus* Pleistocene effects of habitat availability.

In the meantime, we have to remember that inferences about history are susceptible to the general problems of biogeographic testing and the difficulty of assigning confidence [5]. It is essential that data-rich phylogeography, bolstered by emerging sophisticated analytical tools, is not allowed to descend to the domain of story-telling that has marred so much biogeographic ‘analysis’ of this region in the past.

## Figures and Tables

**Table 1:** Information from phylogeography involves two main parameters: the degree of difference among genetic variants (branch length) and the way this diversity is distributed in space.

**Spatial Distribution**	**High Diversity**	**Low Diversity**
Homogenous	deeper coalescence larger population higher gene flow associated with persistent range	shallower coalescence smaller population higher gene flow associated with recent range expansion
Heterogenous	deeper coalescence larger metapopulation reduced gene flow spatial partitioning from older event(s)	shallower coalescence smaller metapopulation reduced gene flow spatial partitioning from more recent event(s)

**Table 2:** Summary of New Zealand invertebrate phylogeography/phylogenetic biogeography within genera, highlighting inferred geophysical process that have left signatures in the distribution and depth of genetic diversity. -Last glacial maximum (LGM) ending 20 k years ago.

**Time**	**Event/Process**	**Invertebrate taxa**	**Pattern/Evidence**
recent	Taupo volcanics	*Acanthophlebia* mayfly[33], *Orthopsyche* caddisfly[34]	Low diversity
		
>2,000		*Hemideina* tree weta[35]	Chromosome contact zone
		
years		*Argosarchus* stick insect[36], *Peripatoides* peripatus[37]	High diversity

LGM	Glaciation of central South Island (beech gap)	*Brachynopus fungus beetle[38], six stonefly genera[39], freshwater snail Potamopyrgus[40]*	Extirpation during a recent glaciation
		
		*Agyrtodes fungus beetle[41]* *cicada M. campbelli [42,43]*	West coast forest refugia Colonization of South Island during glaciation
	
LGM	North and South Islands connected (no Cook Strait)	*Hemideina* tree weta[44,45,46], peripatus[37], *Paranephrops* koura[47], amphipods[48], *Lyperobus huttoni* weevils[49], *Celatoblatta vulgaris* cockroaches[49], *Pachyrhamma edwardsii* cave weta[50], *Epistranus* fungus beetles[51]	Similar or identical haplotypes straddling Cook Strait
		
		*Wainuia* snails[52], *Kikihia* cicadas[53], earthworms[54], *Hemiandrus* ground weta[55], *Talitropsis* cave weta[23,56], *Pristoderus* fungus beetles[51]	Phylogeographic gaps at Cook Strait
	
LGM	South and Stewart Islands connected	freshwater isopods[57], *Kikihia* cicada[58], koura[47]	Similar or identical haplotypes straddling Foveaux Strait
	
LGM	Expanded alpine species	*Wiseana* hepialid moths[58], *Brachaspis* grasshoppers[59], some Kikihia[60] and *Maoricicada* cicadas[43]	Speciation inferred to be within the Pleistocene
	
LGM	Glacial refugia	*Hemideina* tree weta[61], *Clitarchus* stick insects[62,63], *Acanthophlebia* mayflies[33]	Higher diversity in Northland
		
		*Paranephrops* koura[47], *Hemideina* tree weta[45], mite harvestmen[64], *Hemiandrus* ground weta[55], *Potamopyrgus* freshwater snails[40], *Epistranus* fungus beetles[51], *Agyrtodes*[41]	Higher diversity in Nelson region
		
		*Prodontria* scarabaeid beetles[65], *Powelliphanta* snails[66], *Peripatoides* peripatus[67], *Maoricicada* cicadas[42,43], *Niveaphasma* stick insect[68]	Deep lineages in Southland

Pliocene	Southern Alp formation	scree weta *D. Connectens[*69], mite harvestman[64], *Neocicindela[*70]	Intraspecific structure
		
		*Peripatoides* peripatus[49], *Lyperobius* weevils[49], *Celatoblatta* cockroaches[49,56], *Mecodema* beetles[49], *Neocicindela*[70]	Interspecific structure
		
Pliocene	Southern Alp formation	hepialid moths[58], Lycosid spiders[71], Kikihia cicadas[72,73], Sigara water boatmen[74], Sigaus grasshoppers[75], Celatoblatta cockroaches[76], Mecodema beetles[48,77], Lyperobius weevils[49], peripatus[49], 14 species of Maoricicada[78,79]	Radiations attributed to Pliocene uplift
		
		flightless beetles *Prodontria*[65], *Deinacrida* weta[80]	Multiple origins of alpine adaptation
		
		*Paranephrops* koura[47], *Sigara* water boatmen[74], *Brachynopus* fungus beetles[38], *Kikihia* cicadas[60], *Celatoblatta* cockroaches[49,56,76]	Splits across the alps
		
		*Agyrtodes* fungus beetle[41]	Connections across the alps
	
Pliocene	Northland archipelago	*Hemideina* tree weta[61], *Placostylus* snails[81,82], *Clitarchus* stick insects[83], *Amborhytida* snails[84]	Structure concordant with islands
		
		*Paryphanta* snails[84], corophiid amphipods[85]	East-west split (not island concordant)
	
	Three Kings Is	*Placostylus* snails[86], rhytidid snails[84], *Pseudoclitarchus* stick insects[87], *Epistranus* fungus beetles[51]	Divergence from NZ consistent with island age
	
	Campbell Island	freshwater isopods[57]	Divergence from NZ consistent with island age
	
4 MYA	Chatham Island	stag beetles[56,77], cave weta[23,56,77], cockroaches[56,76,77], spiders[71], damselflies[88], cicadas[72,73,89], freshwater isopods[57], amphipods[48]	Divergence from NZ consistent with age of Chatham Islands

Miocene	East Coast Islands	*Hemideina* tree weta (*H. trewicki*)[44,45,46], *Peripatoides* peripatus (*P. morgani*)[37], *Kikihia* cicadas[87]	Divergent clades in Hawkes Bay
	
Miocene	Banks Peninsula volcanic Is	*Hemideina* tree weta (*H.ricta*)[90]	Pleistocene landbridge lead to endemic taxa

**Table 3:** Intraspecific phylogeographic studies of New Zealand invertebrates. Pairwise distances calculated for taxa marked with * using HKY (Hasegawa, Kishino & Yano) from Genbank accessions using Geneious Pro v5.3.4. [112]. Numbers: Indiv.- individuals, Haps.- haplotypes. Regions: N-North Island, S-South Island, C-Chatham Islands. IBD- isolation by distance inferred from correlation between geographic and genetic distance (in most studies a Mantel test was used).

**Taxa**		**Mtdna Sequence**	**Nuclear**	**Number of:**					**Region**			**Maximum Intraspecific Distance**		**Cryptic Species Likely**	**I** **B** **D**	**Patterns**	**Reference**

				**Species**	**Indiv.**	**Locations**	**Haps**	**Environment**	**NI**	**SI**	**CI**						
stick insects	*Acanthoxyla*	COI & COII	ITS sequence	7	33	11	14	forest	N	S		0.022	observed	asexual	no	recent expansion	[103,113]
stick insect	*Argosarchus horridus*	COI & COII	sexual/asexual	1	90	49	46	forest	N	S	C	0.033	Tamura-Nei 1993	no		out of north	[36]
stick insect	*Clitarchus hookeri*	COI & COII	sexual/asexual	1	83/170	30/105	62/99	forest	N	S		0.030	HKY*	no	yes	out of north	[62, 63]
stick insect	*Niveaphasma annulata*	COI	EF1-alpha sequence	1	97	66	48	subalpine/forest		S		0.044	HKY+ Γ+I	no	yes	glaciation causing isolation	[68]
grasshopper	*Brachaspis nivalis*	COI		2	26	13	22	subalpine/open		S		0.106	K2P	yes		regional	[59, 114]
grasshopper	*Sigaus australis*	COI & 12S		1	130	22	32	subalpine		S		0.083	uncorrected	yes		regional	[75, 114]
grasshopper	*Sigaus piliferus*	COI		1	51	13	31	montane	N			0.064	K2P	yes		regional	[114]
weta (cave)	*Talitropsis sedilloti*	COI & COII		1	56	43	35	forest	N	S		0.031	uncorrected	no	no	out of south	[23, 56, 77]
weta (giant)	*Denacrida connectens*	COI	allozymes	1	78	24	40	subalpine		S		0.130	GTR+Γ+I	no		regional	[69, 115, 116]
weta (ground)	*Hemiandrus maculifrons*	COI	morphology	2	41	24	41	forest	N	S		0.120	uncorrected	yes	yes	out of south	[55, 117]
weta (ground)	*Hemiandrus pallitarsis*	COI	drumming & morphology	1	88	18	55	forest	N			0.089	TVM+ Γ+I	no	no	out of south	[55, 118]
weta (stone)	*Hemideina maori*	COII		1	27	10	13	subalpine		S		0.055	Tamura-Nei 1993	no		regional	[90]
weta (tree)	*Hemideina crassidens*	COI	allozymes &chromosomes	1	12	12	12	forest	N	S		0.127	HKY+I	no		out of Nelson	[44, 46]
weta (tree)	*Hemideina thoracica*	COI &12S	allozymes & chromosomes	1	191	49	60	forest	N			0.095	uncorrected	no	no	out of north	[35, 109]
beetle	*Agyrtodes labralis*	COI		1	187	79	97	forest		S		0.056	HKY*			expansion across alps west to east	[41]
beetle	*Brachynopus scutellaris*	COI		1	113	34	73	forest		S		0.060	HKY*		yes	north/south glaciation gap	[38]
beetle	*Epistranus lawsoni*	COI		1	168	78	116	forest	N	S		0.246	uncorrected	yes	yes	spatially & temporally continuous	[51]
beetle	*Hisparonia hystrix*	COI		1	105	39	47	forest		S		0.018	HKY*	no	yes	north/south glaciation gap	[38]
beetle	*Pristoderus bakewelli*	COI		1	88	53	77	forest	N	S	C	0.149	uncorrected	yes	no	spatially & temporally continuous	[51]
cicada	*Kikihia subalpina*	COI & COII	song	1	114	79	58	montane	N	S		0.035	HKY+I	no		North I vs South I/out of Nelson	[59]
cicada	*Kikihia muta*	COI & COII	song & colour	1	162	88	107	open	N	S	C	0.067	uncorrected	yes		regional	[89]
cicada	*Maoricicada campbelli*	COI & A6-A8	song	1	212	91	95	open	N	S		0.066	HKY+I	no		out of north and southern refuge	[42, 43]
stonefly	*Zelandoperla decorata*	COI	H3 sequence	1	144	63	45	aquatic		S		0.024	HKY	no		recent expansion/gene flow	[105]
stonefly	*Zelandoperla fenestrata*	COI	H3 sequence	1	186	81	71	aquatic		S		0.091	Tamura-Nei 1993	no		regional	[105]
mayfly	*Acanthophlebia cruentata*	Cytb		1	186	19	34	aquatic	N			0.037	HKY*	no	yes	out of Northland, Taupo extinction	[33]
caddisfly	*Orthopsyche fimbriata*	COI		1	157	16	23	aquatic	N			0.047	HKY*	no	yes	Taupo extinction?	[34]
damselfly	*Xanthocnemis zealandica*	COI	allozymes	1	27	15	13	aquatic	N	S	C	0.012	uncorrected	no		recent expansion/dispersal	[88]
waterboatman	*Sigara potamius*	COI		1	35	28	24	aquatic		S		0.071	HKY*			east vs west/regional	[74]
amphipods	*Paracalliope fluviatilis*	COI	allozymes	1	54	14	17	aquatic	N	S		0.260	uncorrected	yes		regional, out of south?	[107]
isopod	*Austridotea lacustris*	COI	ITS	1	24	12	19	aquatic		S	C	0.113	HKY			dispersal to Chathams	[57]
mite havestmen	*Aoraki denticulata*	COI	morphology	1	119	17	84	forest		S		0.192	uncorrected	yes		out of Nelson?	[64]
centipede	*Craterostigmus crabilli*	COI & 16S	18S & 28S sequence	2	14	9	13	forest	N	S		0.325	HKY*	yes		out of Nelson	[104]
peripatus	*Peripatoides sympatrica*	COI	allozymes	1	41	14	16	forest	N			0.027	GTR+Γ+I	no		little pattern	[37, 108]
peripatus	*Peripatoides* n. sp.	COI		1	47	21	18	forest		S		0.110	K2P	yes		regional	[67]
koura	*Paranephrops planifrons*	COI		1	62	43	62	aquatic	N	S		0.135	GTR+Γ+I	yes	yes	out of Nelson northwards	[47]
koura	*Paranephrops zealandicus*	COI		1	43	33	39	aquatic	N	S		0.227	GTR+Γ+I	yes	yes	regional/out of Nelson souththwards	[47]
snail	*Potamopyrgus antipodarum*	CytB	Microsats. for ploidy	1	638	20	45	aquatic	N	S		0.037	GTR+I	no	yes	out of north	[40]

**Table 4:** Exemplars of New Zealand invertebrate phylogeography studies matching hypothetical extremes in terms of depth of diversity and patchiness of diversity. See Tables 2 and 3 for additional detail.

**Spatial Distribution**	**High diversity**	**Low diversity**
Homogenous	Beetle- *Epistranus lawsoni* Spiders- *Anoteropsis* species	Stick insect- *Acanthoxyla* Damselfly - *Xanthocnemis zealandica* stonefly - *Zelandoperla decorata*
Heterogenous	Regional Cicada- *Maoricicada campbelli* Weta- *Deinacrida connectens* Koura- *Paranephrops*	Regional Cicada- *Kikihia subalpina* beetle- *Hisparonia hystrix* Graded Stick insect- *Clitarchus hookeri*
